# Public health impact and harm reduction implications of xylazine-involved overdoses: a narrative review

**DOI:** 10.1186/s12954-023-00867-x

**Published:** 2023-09-12

**Authors:** David T. Zhu

**Affiliations:** Medical Scientist Training Program, School of Medicine, Virginia Commonwealth University, 1201 E Marshall St, Richmond, VA 23298 USA

**Keywords:** Xylazine, Opioid, Overdose, Harm reduction, Public health, Puerto Rico, USA, Canada

## Abstract

**Introduction:**

Xylazine, an α2-adrenoceptor agonist sedative commonly used in veterinary medicine, is not approved for human use. Nevertheless, xylazine-involved overdose rates have surged in recent years, fueled by an increasingly toxic and synthetic illicit drug supply in North America.

**Methods:**

This narrative review assessed major epidemiological trends in xylazine-involved overdoses in North America, aiming to identify harm reduction priorities. A literature search was conducted using four bibliographic databases (PubMed, Scopus, Embase, and ScienceDirect) and three preprint servers (medRxiv, bioRxiv, and Europe PMC) on May 28, 2023, to capture articles related to combinations of keywords such as “xylazine”, “opioid”, and “harm reduction”.

**Results:**

Xylazine emerged as an adulterant in Puerto Rico in 2001, likely diverted from veterinary supplies. By the mid-2010s, it began proliferating across unregulated US drug markets, often contemporaneously with illicitly manufactured fentanyl (IMF), displaying characteristics of a syndemic. Initially concentrated in Northeastern regions (e.g., Philadelphia, Connecticut, Maryland), xylazine-involved overdoses later extended to the Rust Belt, Southern, and Western regions of the USA in the late 2010s and early 2020s. During this time, xylazine-involved overdoses also surged in Canada, particularly in Western provinces (British Columbia and Alberta) and Ontario with established IMF-dominated unregulated drug markets.

**Discussion:**

Over the past two decades, xylazine-involved overdoses have been rapidly rising in North America and exhibit few signs of slowing down, representing a serious public health epidemic. Numerous factors may have contributed to this, including limited epidemiological surveillance and drug checking for xylazine and emerging novel adulterants; further, barriers to comprehensive, trauma-informed, non-stigmatizing treatment and social services have also exacerbated this issue. While several epidemiological and ethnographic studies have assessed these factors in the USA, limited evidence is available in Canada where xylazine emerged more recently. This underscores the need for additional research and harm reduction measures.

**Conclusion:**

Harm reduction-informed public health guidelines and programs are urgently needed to promote a safer supply, strengthen the healthcare system capacity to prevent and respond to xylazine-involved overdoses, and address social and structural disparities in health outcomes.

**Supplementary Information:**

The online version contains supplementary material available at 10.1186/s12954-023-00867-x.

## Introduction

The North American opioid crisis is rapidly expanding, leading to unprecedented levels of morbidity and mortality among people who use drugs (PWUD) and placing an immense strain on the healthcare system [1]. In 2022, there were more than 108,000 estimated drug overdose deaths, of which approximately three-quarters involved opioids, marking the deadliest year of this epidemic [2]. A major factor contributing to this is the ever-shifting and increasingly toxic drug supply [3, 4]. The USA has borne witness to four overlapping “waves” of opioid supply shifts: the widespread proliferation of prescription opioids during the 1990s; followed by heroin’s rising dominance starting in 2010; the emergence of illicitly manufactured fentanyl (IMF) saturated unregulated drug markets around 2013; and, most recently, a polysubstance crisis ushering in new generations of highly potent, synthetic adulterants such as xylazine [3–5].

Xylazine is a non-opiate sedative, analgesic, and muscle relaxant that shares its drug class (α2-adrenoreceptor agonists) with medications such as clonidine, lofexidine, tizanidine, and dexmedetomidine [6]. It was initially developed as an antihypertensive agent by Farbenfabriken Bayer AG in 1962; however, subsequent testing revealed severe adverse events related to hypotension and central nervous system (CNS) depression [6, 7]. Consequently, xylazine never gained approval for human use; however, it was approved by the US Food and Drug Administration (FDA) in 1972 exclusively for use in veterinary medicine [6, 7]. At present, xylazine remains unregulated under both the Controlled Substances Act (CSA) in the USA [8] and the Controlled Drugs and Substances Act (CDSA) in Canada [9].

Fatal xylazine-positive overdoses, often co-occurring with synthetic compounds such as IMF and its analogs, surged dramatically in the past decade in North America. These overdoses have increased approximately 12-fold between 2018 and 2021 in the USA [6]. Importantly, naloxone—an opioid antagonist medication that is safe and effective for reversing opioid-induced respiratory depression during overdose—does not directly address the effects of xylazine as it is not an opioid, thereby introducing new challenges regarding overdose response best practices within clinical- and community-based settings [8, 10]. Altogether, this pressing issue has prompted The White House Office of National Drug Control Policy (ONDCP) to designate fentanyl associated or adulterated with xylazine (FAAX) as an emergent health threat to the USA in April 2023 and issue a comprehensive response plan in July 2023 [11, 12]. Similar cases are also on the rise in Canada and other countries such as the UK, marking the first xylazine-involved overdose outside of North America [9, 13]. Against the backdrop of this global health crisis, it is imperative to renew efforts in delivering evidence-based public health and harm reduction programs to facilitate secondary and tertiary prevention of adverse health outcomes following xylazine exposure.

Various reviews exist on the clinical manifestations and pharmacokinetics of xylazine poisonings, including case reports and toxicological studies [13–19]. However, to the best of my knowledge, there are no reviews that have assessed the public health impact and harm reduction implications of the xylazine epidemic in North America. This narrative review aims to fill this research gap by outlining (1) the emergence of xylazine in the illicit drug supply, (2) spatiotemporal trends in xylazine-involved opioid overdoses in North America, and (3) current challenges and opportunities for harm reduction programs and community-based initiatives to address this growing crisis.

## Methods

A literature search was conducted in four bibliographic databases (PubMed, Scopus, Embase, and ScienceDirect) and three preprint servers (medRxiv, bioRxiv, and Europe PMC) to capture peer-reviewed articles and gray literature. Various combinations of search terms such as “xylazine”, “Rompum”, “AnaSed”, “Sedazine”, “opioid”, “opiate”, “overdose”, “harm reduction”, “Puerto Rico”, “Mexico”, “United States”, and “Canada”, were used to retrieve articles on May 28, 2023. The full search strategy, including combinations of MeSH terms and Boolean operators, is described in Additional file 1: Table S1. Backward reference searching was performed when indicated, notably, to facilitate in-depth investigations of specific harm reduction organizations addressing the xylazine crisis. Inclusion criteria were English-language primary articles or reviews that evaluated the epidemiology or public health impact of xylazine in North America. Both quantitative and qualitative studies were included. However, case reports were excluded as the primary focus of this review was the public health and harm reduction implications, rather than the clinical manifestations, of xylazine-involved opioid overdoses.

## Results

The previously specified search and screening criteria yield 2999 articles; after deduplication, 2535 articles remained. A total of 19 peer-reviewed articles discussing relevant public health and harm reduction implications of the emerging IMF-xylazine crisis fit the eligibility criteria. [6, 13–30]. In addition, 22 gray literature articles were identified through backsearching of the captured articles, including xylazine reports from federal government and public health agencies (*n* = 9) [2, 5, 7–9, 11, 12, 31, 32], state and local public health agencies (*n* = 8) [33–40], organization websites (*n* = 3) [41–43], conference abstracts (*n* = 1) [34], and news sources (*n* = 1) [44].

### Emergence of xylazine in Puerto Rico

The first known illicit human consumption of xylazine, known colloquially as *anestecia de caballo* (“horse anesthesia”), was documented in Puerto Rico in 2001, identified through surveillance and laboratory testing conducted by the US Drug Enforcement Administration (DEA) [6]. Over the next decade, a steady rise in xylazine-involved overdoses was observed across Puerto Rico, concentrating in cattle-farming communities wherein it was likely diverted from veterinary sources and sold on unregulated drug markets [20, 21]. For instance, an early study that analyzed used syringes across 11 different municipalities in Puerto Rico, utilizing gas chromatography-mass spectrometry (GC/MS) analysis, estimated an aggregate xylazine prevalence of 37.6%, contrasting with remarkably higher estimates (98–100%) in the west-central cattle-farming towns of Arecibo, Yauco, and Guanico [20]. A later study estimated a substantially higher xylazine prevalence of 80.7% [22]. Injection was the primary mode of xylazine administration (84.5%), followed by inhalation (14.1%) and smoking (1.4%) [22].

### Proliferation of xylazine in Puerto Rico

Numerous factors have facilitated xylazine’s spread across Puerto Rican unregulated drug markets. Firstly, xylazine may have allured illicit drug manufacturers on the island due to its capacity to compensate for the shortcomings of other substances, such as IMF [20, 22]. Ethnographic studies suggest that, by incorporating xylazine into polydrug formulations, it could subjectively prolong the euphoric effects of compounds such as IMF, thereby extending IMF’s ‘short legs’ and temporarily alleviate craving or withdrawal symptoms [6, 21]. As a result, xylazine became actively sought after by a considerable number of PWUD [6, 21].

Xylazine was most commonly sold in the form of a "speedball" or "goofball", where it was combined with stimulants (e.g., cocaine or amphetamines) and opioids (e.g., heroin or IMF) [20, 22]. In a mixed-methods study, the prevalence of xylazine-containing speedballs in 2007 was estimated to be 42.3% [22], a figure possibly underestimated due to recall bias or social desirability bias. Other studies reported higher prevalence estimates for xylazine-positive speedballs. Notably, a study from 2005 to 2007 employing GC/MS showed a higher proportion of xylazine-positive syringes among individuals who use speedballs (90.6%) compared to those who did not (66.7%) [20]. Additionally, another study documented that speedball users had 9.34 times higher odds of using xylazine than non-speedball users [22].

Secondly, another factor contributing to its proliferation was the appeal it offered in terms of market differentiation. By mixing xylazine with various concentrations and types of other substances, illicit suppliers could distinguish their specific "stamp" (brand) from competitors and attract additional customers [21]. Some PWUD favored pre-mixed formulations containing xylazine, while others preferred non-premixed products, which allowed them to adjust the ratio of xylazine to opioids according to their personal preferences [21]. Noteworthy combinations included *el regalito* (“the small gift”), comprising approximately one-fifth heroin and four-fifths xylazine, as well as *el combito* (“the small combo”), a speedball blend of heroin, cocaine, and xylazine, obtainable for US$8 [21]. The ever-shifting landscape of the illicit drug supply, coupled with the diverse array of polydrug concoctions, underscores the imperative to enhance ongoing epidemiological surveillance and monitoring of emerging adulterants, such as xylazine, to inform harm reduction interventions.

Thirdly, unintentional exposure to xylazine was distressingly common among PWUD. Some PWUD mistakenly believed that they could identify the presence of xylazine based on visual cues, as well as the taste and odor of drug mixtures [22]. However, this approach proved to be unreliable due to considerable variation in the physical appearance of xylazine-containing substances, likely due to the presence of diverse cutting agents and adulterants, ranging from different colors and forms such as powders, pebbles, and crystals [22]. Notably, among people who self-described xylazine use in Puerto Rico, only approximately 49% of the syringes contained the drug as confirmed by GC/MS [22]. Further, approximately 22% of the syringes from people who claimed not to have used xylazine actually contained it, indicating that many PWUD were unknowingly exposed to xylazine [22]. These findings highlight the need for improving education and public awareness about the widely varying appearance of xylazine-containing mixtures, as well as expanding rapid point-of-care testing (POCT) technologies and drug checking services to inform PWUD about xylazine and other adulterants in their supply.

Fourthly, inadequate epidemiological surveillance infrastructure, coupled with inaccessible drug checking and treatment services, likely exacerbated the spread of xylazine in Puerto Rico. For instance, limited xylazine surveillance underestimated its true prevalence, impeding a proportionate public health response to the emerging crisis [6]. Moreover, hospitals were ill-equipped and insufficiently trained to provide effective clinical care for people who chronically used xylazine. These individuals often presented with severe withdrawal symptoms and soft tissue injuries, including necrotic skin ulcers which did not always appear at injection sites; in extreme cases, amputation of affected extremities became necessary [6, 19, 21]. Further, many xylazine patients also experienced pervasive stigma from healthcare providers; this stigma has been described as being “compounded” due to the visual appearance of xylazine-related wounds, as well as its strong odor, further alienating these individuals from the healthcare system [21]. Ultimately, left underdetected and undertreated, xylazine-involved overdoses surged at an alarming rate in Puerto Rico, encountering little resistance and limited public health and harm reduction interventions. The early Puerto Rican xylazine epidemic highlights the urgent need for multi-sectoral collaborations among healthcare providers and harm reduction organizations to address structural barriers in access to timely, trauma-informed, non-stigmatizing treatment and supportive care for people affected by xylazine.

### Emergence of xylazine in the USA

The movement of xylazine from Puerto Rico into the mainland USA may be attributed to two primary mechanisms: the need for specialized health services to manage xylazine-related injuries among PWUD [21, 23, 24] and the broader influence of illicit drug market dynamics [23, 24]. As aforementioned, many xylazine patients faced obstacles in accessing effective treatment in Puerto Rico, exacerbated by stigma from healthcare providers [21]. As a result, local government authorities in Puerto Rico relocated many xylazine patients to northeastern US regions such as Philadelphia, where they received care from largely unregulated treatment services, lacking the guidance of evidence-based best practices [21, 23]. This relocation may have created a demand for the drug in these new locations, creating an opportunity for illicit suppliers to introduce xylazine into the local drug market [21]. Moreover, even prior to the earliest official entrance of xylazine in the USA in 2006, ethnographic observations unveil that there was already an established early demand for xylazine as an adulterant in the open-air drug markets in predominantly Puerto Rican, segregated, low-income neighborhoods in Philadelphia, which is recognized as the earliest epicenter of this epidemic [23]. Puerto Rican drug distributors in Philadelphia who were intimately connected with their counterparts in Puerto Rico may have facilitated the entry of xylazine into these new illicit drug markets [23, 24]. These demand- and supply-side factors thus laid the groundwork for the emergence and subsequent proliferation of xylazine across the USA.

### Epicenters of xylazine-involved overdoses in the USA

During the transformative period of the mid- to late-2010s, xylazine began proliferating across unregulated drug markets in the northeastern USA [6, 23]. Among the first epicenters of this emerging crisis were Philadelphia, Maryland, and Connecticut [6, 25]. Philadelphia witnessed a gradual increase in heroin- and IMF-related overdoses involving xylazine between 2010–2020 (rising from less than 2% between 2010 and 2015, 7% between 2016 and 2017, 14% in 2018, and 26–31% between 2019 and 2020), followed by a sharp increase by mid-2021 when such overdoses soared to an alarming 90% [6, 23–25]. The proliferation of xylazine in Maryland and Connecticut began slightly later than in Philadelphia. Interestingly, both states exhibited similar rates of xylazine-involved opioid overdoses, possibly due to similar drug trafficking routes and similar drug compositions within heroin- and IMF-dominated unregulated drug markets [6, 23]. Between 2017 and 2018, such overdoses were rare (0.2–1.1% in Maryland and 0% in Connecticut), followed by a steady rise in 2019 (4.3% in Maryland and 3.8–5.8% in Connecticut), 2020 (12.5–17% in Maryland and 10.2–11.4% in Connecticut), 2021 (19.3% in both Maryland and Connecticut), and 2022 (24.7% in Connecticut) [23, 25, 27, 33, 34]. No publicly available data were available for Maryland in 2022 at this time of writing. Further, the highest rates of xylazine-involved overdose deaths in Connecticut appeared to occur in cities such as Windham, New London, and Hartford, while in Maryland these predominantly occurred in Baltimore [33, 34].

### Proliferation of xylazine in the USA

Consistent with the observations made in Puerto Rico, IMF was nearly ubiquitously found in xylazine-positive overdoses in the USA, often in greater than 90% of cases, fueling the spread of xylazine (which tends to penetrate existing IMF-dominated unregulated drug markets). This provides further evidence of their strong ecological link and syndemic nature, exacerbating existing vulnerabilities such as PWUD’s lack of access to healthcare services, as well as limited surveillance and drug checking [23, 24, 45–47]. In a recent study of 21 jurisdictions within the CDC's State Unintentional Drug Overdose Reporting System (SUDORS), monthly rates of fentanyl mixed with xylazine (FMX) overdose deaths rose by nearly fourfold (from 2.9 to 10.9%) between January 2019 and June 2022 [46]. During this period, Maryland (27.7%), Connecticut (26.4%), and Pennsylvania (23.3%) had the highest monthly rates of fatal FMX overdoses [28]. Later studies in 2021–2022 reveal even higher co-occurrence rates, with approximately 90% and 80.5% of IMF-positive overdoses involving xylazine in Philadelphia and Maryland, respectively, and an astounding 99.3% of xylazine-positive overdoses involving IMF in Connecticut [23, 25, 27]. The Georgia Department of Public Health estimated that 100% of xylazine poisonings co-involved IMF in 2022 [35]. Aside from IMF, other common co-occurring substances in xylazine-positive overdoses include cocaine (35.2%), methamphetamine (18.0%), alcohol (14.9%), heroin (14.6%), prescription opioids (14.3%), and benzodiazepines (13.5%) [28]. Overall, these concerning trends warrant more comprehensive monitoring and epidemiological surveillance to better understand the rising prevalence of xylazine in illicit drug supplies and its impact on overdoses. Additional research is needed to further clarify xylazine’s mechanisms of action and role in polysubstance overdoses.

In the late 2010s and early 2020s, the spread of xylazine gained momentum, reaching other Northeastern cities and extending to the Rust Belt, Southern, and Western US census regions [6, 23, 24]. Again, this paralleled the trajectory of IMF, reinforcing their syndemic nature [23, 24, 45–47]. Between January 2019 and June 2022, xylazine-involved overdoses were most frequent in the Northeast (49.9%), followed by the Southern (32.0%), Midwestern (17.0%), and Western (1.1%) US census regions [28]. Notably, these regional trends varied over time. From 2020 to 2021, the DEA observed a surge in laboratory identifications of xylazine in Southern US census regions (193%), closely followed by the Western (112%), Northeastern (61%), and Midwestern regions at a comparatively modest 7% [8]. Concurrently, the rise in xylazine-involved overdoses was most pronounced in the Southern US census regions (1127%), followed by the Western (750%), Midwestern (516%), and Northeastern regions (103%) [8]. By 2021, Southern regions surpassed the Northeastern regions in both xylazine identifications and xylazine-involved overdoses, while Midwestern regions fell behind the Western regions in terms of xylazine identifications but still had more xylazine-involved overdoses than the Western regions [8]. Georgia emerged as one of the most heavily impacted Southern states, as evidenced by the steep increase in xylazine-involved overdoses from 0.8 to 9% between 2020 and 2022 [35]. Of particular concern, the incidence of xylazine-related deaths in Georgia surged by a staggering 1120%, a stark contrast to the modest 12% increase in overall drug-related deaths during the same period [35]. In the Western regions, Oregon and Nevada also had notably higher rates of FMX overdose deaths [31, 36–38].

These epidemiological trends may be attributed to numerous factors, including increased IMF penetration in the illicit drug supply (thereby providing a wider market for xylazine infiltration), unintentional exposure to xylazine, and barriers to the implementation of harm reduction and public health interventions [23, 24]. For instance, between 2019 and 2022, Oregon's high-intensity drug trafficking areas (HIDTA) reported a staggering 12-fold rise in seizures of counterfeit pills containing fentanyl, accompanied by a 74% increase in fentanyl-related overdose deaths, expanding the market for xylazine involvement [36].

Akin to the circumstances witnessed in Puerto Rico, unintentional exposure to xylazine surfaced as a common occurrence in the USA. While some PWUD actively sought out formulations containing xylazine, others actively attempted to avoid it due to severe withdrawal symptoms, as well as the potential for soft tissue injuries such as necrotic skin ulcers, which are hypothesized to arise from localized tissue hypoxemia [6, 23, 26]. Moreover, the sedative effects of xylazine rendered PWUD more susceptible to theft or assault while under its influence, providing yet another reason for many PWUD to avoid xylazine [23, 26]. Importantly, 85.8% of PWUD seeking opioids were unwittingly exposed to xylazine in Maryland [25]. Among those intending to purchase "fentanyl only," "heroin only," or a combination of "fentanyl and/or heroin," xylazine was detected in 90.9%, 20.0%, and 84.3% of samples, respectively, though it should be noted that the sample size for "heroin only" was small (*n* = 5), limiting the validity of this estimate [25]. These findings underscore the importance of comprehensive drug testing, vigilant monitoring of xylazine's co-involvement with other substances such as IMF, establishing cross-sectoral collaborations to improve access to harm reduction supplies such as xylazine test strips, and widespread public health messaging to assist PWUD in minimizing unintended exposure.

### Social and demographic characteristics of people who use xylazine in the USA

Regarding the social determinants of health, numerous studies have consistently found that males and non-Hispanic White populations are disproportionately impacted by the emerging xylazine crisis. Xylazine-involved opioid overdose deaths in Connecticut were more prevalent among males (80.9%), non-Hispanic White individuals (74.0%), Hispanic/Latino individuals (17.1%), and non-Hispanic Black individuals (6.8%) [27]. Likewise, in Maryland, such overdoses were more prevalent among non-Hispanic White populations compared to non-Hispanic Black populations (18.7% vs. 15.9%), as well as those aged between 25 and 55 and involved in injecting drugs [34]. Findings from the Georgia Department of Public Health indicate that xylazine-involved drug overdose deaths were highest among males (71%), 131% higher among non-Hispanic White individuals compared to non-Hispanic Black individuals, and 121% higher among Hispanic individuals compared to their non-Hispanic counterparts [35]. These racial and ethnic trends are generally consistent with SUDORS data showing higher occurrences among males (72.9%), non-Hispanic White (65.6%), non-Hispanic Black (23.9%), and Hispanic/Latino (9.2%) populations [28]. Further epidemiological research is warranted to more comprehensively evaluate the social and structural determinants of xylazine-involved overdoses in North America.

### Emergence, epicenters, and proliferation of xylazine-involved overdoses in Canada

Xylazine began to proliferate in Canada around 2019, although a few xylazine-involved overdoses were sporadically detected as early as 2012 [9]. Western regions, namely British Columbia (BC) and Alberta, and Ontario are among the earliest epicenters of this crisis. In 2019, Health Canada’s Drug Analysis Service (HC DAS) reported that 67.3% of the xylazine-containing samples in Canada came from Alberta, 28.3% from BC, and 3.4% from Ontario [9]. By 2022, the prevalence of such samples considerably decreased to 2.5% and 19.3% in Alberta and BC, respectively, while it rose to 74.9% in Ontario [9]. Most xylazine-containing samples in Ontario were identified in Toronto (24% in 2021, dropping to 13% in 2022) [31].

These three provinces tend to have the highest opioid overdose deaths (cumulatively 87% of all accidental apparent opioid toxicity deaths in Canada), suggesting that xylazine may have entered Canada by penetrating established unregulated drug markets wherein illicit sales of synthetic opioids such as IMF are commonplace. Between 2019 and 2022, the age-adjusted opioid overdose mortality rate (per 100,000 population) increased in BC (from 19.9 to 44.0), Alberta (from 14.1 to 32.6), and Ontario (from 10.8 to 16.7) [32]. The surge in IMF-related overdoses in these provinces may have fueled the rising prevalence of xylazine in these regions between 2019 (ranging from 56 to 89%) and 2021 (ranging from 85 to 92%) [32]. While Yukon and the Northwest Territories also witnessed notable changes in IMF-involved overdoses, few xylazine cases were documented in these areas [32], possibly due to their geographical isolation and relatively smaller population size, which may result in different drug trafficking patterns and less exposure to xylazine-contaminated drug supplies compared to more densely populated regions. Data from HC DAS indicate that the proportion of IMF samples containing xylazine increased from 1.4 to 6.9% between 2020 and 2022 [9]. Reports indicate that many IMF-positive samples also contained xylazine. However, caution should be exercised in interpreting the limited data available in Canada regarding the underlying factors contributing to the emergence and spread of xylazine in the unregulated drug market and supply. Further research, particularly robust public health investigations and in-depth ethnographic studies, is needed to shed light on the complex dynamics around xylazine's emergence and subsequent proliferation across Canadian illicit drug markets and to inform evidence-based harm reduction strategies.

The Canadian Community Epidemiology Network on Drug Use (CCENDU) estimates that the majority of xylazine samples in Canada (79%) contain two to four co-occurring substances, with IMF being most commonly found in combination with xylazine (93%) [31], aligning with HC DAS estimates in the range of 90–98% [9]; these figures are similar to the USA. Contemporaneous use of benzodiazepine mixed with xylazine is also common in both the USA (28.4%) and Canada (HC DAS estimates it at 28%, while CCENDU estimates place it much higher at 57–61%) [9, 29, 37]. Methamphetamine was the least frequently observed co-occurring substance with xylazine in both countries, being implicated in only 10.0% of xylazine-present overdose deaths in the USA and 5.1% in Canada [9, 23]. However, several notable differences exist between the USA and Canada. For instance, xylazine-cocaine formulations are substantially less prevalent in Canada (3.6%) than in the USA (30.9%) [9, 28]. Additionally, in the USA, heroin is commonly co-present with xylazine (28.4%), whereas in Canada, this is not the case (only 4.3%). These differences are important as xylazine is frequently found within a speedball mixture involving IMF, heroin, cocaine, etc., in both the USA and Puerto Rico; perhaps these combinations are less frequent in Canada. Further research is warranted to better understand the unregulated drug market dynamics that influence these trends. There are limited national-level data on xylazine seizures and poisonings in Canada, impeding a comprehensive evaluation of spatiotemporal trends in xylazine-involved opioid overdoses as well as how the Canadian xylazine crisis might differ from that of the USA. Variations in regional drug preferences, trafficking routes, and market dynamics may contribute to the observed differences between Canada and the USA and warrant further research.

## Discussion

To the best of my knowledge, this is the first review article centered on the public health implications of the emerging xylazine crisis. This narrative review outlines major epidemiological trends in xylazine's emergence and proliferation within unregulated drug markets across Puerto Rico, the mainland USA, and Canada, as well as its increasing implication in polysubstance overdoses. Various factors have driven the surge in xylazine-involved overdoses over the past two decades, including FMX syndemic interactions that exploit health vulnerabilities and structural barriers among PWUD, limited access to comprehensive and continued care for xylazine-related injuries, stigma from healthcare providers, insufficient epidemiological surveillance and availability of drug checking programs, and barriers to harm reduction and public health interventions to tackle this problem [23, 24, 45–47].

Significant gaps in epidemiological surveillance and drug checking programs exist across jurisdictions. Since xylazine is a relatively new and emerging adulterant in opioid-involved overdoses across North America, lack of awareness and standardized surveillance procedures may lead to underreporting or misclassification of xylazine-involved overdoses. Further, chronically under-resourced medical examiner/coroner offices across the USA and Canada often lack the funding, institutional support, and guidelines to comprehensively implement standardized and routine toxicological testing for xylazine [48]. Nationwide efforts by the CDC and the Public Health Agency of Canada to monitor trends in xylazine-involved overdoses are complicated by obstacles such as limited data on overdose profiles (e.g., lack of data on the concentrations and types of drugs involved in xylazine-involved polysubstance overdoses), insufficiently precise ICD-10/ICD-10-CM coding of xylazine, and delayed data availability which hinders the widespread dissemination of real-time information on xylazine-involved cases that is essential to guiding harm reduction and public health interventions [6, 8, 9, 21, 23, 24].

It should be noted that some apparent spatiotemporal trends in xylazine-involved overdoses might partially reflect changes in drug checking and surveillance rather than the migration of xylazine itself. For instance, the three apparent epicenters of xylazine in the USA (Philadelphia, Connecticut, and Maryland) began routine toxicological testing for xylazine earlier than many other cities and states in the USA, which may partially explain their relatively higher rates [30, 33, 39]. The White House Office of National Drug Control Policy’s (ONDCP) recent response plan to the emerging FMX crisis highlights several key strategies to address these drug surveillance and monitoring issues to ensure accuracy and precision. In particular, The White House ONDCP’s recommendations to widely implement guidelines for routine toxicological and forensics testing of xylazine-involved overdoses, standardize the use of diagnostic ICD-10/ICD-10-CM codes, and strengthen community-based testing of xylazine should be emphasized to ensure that PWUD have timely and reliable access to information about their drug supplies [12].

The White House ONDCP's strategy to empower community-based stakeholders underscores the important role of harm reduction organizations and local agencies engaged with drug checking in the emerging FMX crisis [12]. Rapid point-of-care testing (POCT) and xylazine test strips should be made broadly available for PWUD and can be distributed by harm reduction organizations [41, 42]. In Philadelphia, the Center for Forensic Science Research and Education (CFSRE) found that some xylazine test strips demonstrate high sensitivity (100%), specificity (85%), and precision (91%), underscoring the importance of widely disseminating this crucial harm reduction resource to PWUD at-risk of experiencing a xylazine-involved overdose [41, 42]. However, these test strips have been shown to yield false positives when co-occurring with certain substances (e.g., diphenhydramine, lidocaine, levamisole, and MDMA) and are comparatively more expensive than fentanyl test strips [41, 42, 44]. Additional funding and support is needed to ensure widespread community-based distribution of xylazine test strips to overcome barriers to implementation. Accessible community-based drug checking programs should be expanded alongside public health and harm reduction messaging to provide real-time information to PWUD about the contents of their drug supplies; such messaging should focus on highlighting the toxicity of xylazine and encourage risk-reduction practices to mitigate unwanted exposure to xylazine, with caution exercised to avoid stigmatizing people who have used xylazine (e.g., avoiding potentially harmful labels such as “zombies”) and avoid fueling moral panic-driven discourses.

Community-driven solutions to the emerging xylazine crisis should strive to provide holistic services that address the upstream social and structural factors contributing to xylazine-involved overdoses. For instance, in Philadelphia, Savage Sisters is a nonprofit organization that provides food, housing, and social services to individuals affected by xylazine [43]. Local harm reduction champions are imperative to build trust and social capital with communities affected by diseases of despair and may serve as effective re-entry points into the healthcare system [49, 50]. Further, community-based stakeholders have the potential to provide culturally-sensitive, trauma-informed, non-stigmatizing, and more tailored solutions to local community needs for patients with opioid use disorders (OUD), and therefore, should be leveraged on a wider scale to address the emerging xylazine crisis [40].

Additionally, other harm reduction strategies, such as naloxone and supplemental oxygen equipment, should be broadly disseminated to support respiration during an overdose [6]. However, it should be noted that naloxone, an opioid antagonist medication, can reverse respiratory depression from co-occurring opioids in polysubstance overdoses (e.g., IMF or heroin) but does not directly address the risks associated with xylazine as it is a non-opioid sedative [6, 10]; thus, further research and technological innovation is needed to develop novel xylazine reversal agents, and community-based harm reduction initiatives should be leveraged to widely distribute these supplies.

Although this review primarily focused on the public health implications of xylazine-involved overdoses, rather than the clinical management of xylazine-related injuries, it is important to highlight that significant disparities exist with regard to access to timely and effective treatment services. Severe withdrawal symptoms and xylazine-related wounds (necrotic skin ulcers) may require more intensive or specialized courses of treatment [6, 21, 23]. Therein lies an urgent need for hospitals and harm reduction organizations to allocate more resources for xylazine care and implement up-to-date evidence-based, harm reduction-informed guidelines for the effective clinical management of patients who use xylazine. Further, healthcare systems should consider recruiting community-based stakeholders (e.g., mobile clinics) to narrow disparities in access to care [6].

Additionally, stigma is a major concern. Early ethnographic findings from Puerto Rico indicate that people who use xylazine experience significant stigma, which may be compounded due to the severe visual appearance and odor of xylazine-related wounds [21]. This stigma is highly problematic as it may lead individuals who have used xylazine to delay, avoid, or leave care prematurely. Disruptions to continued xylazine care may lead to potentially worsened health outcomes [6, 21]. Clinical and public health interventions should focus on addressing shortages of addiction specialists and funding, challenging discriminatory attitudes and implicit biases, and providing trauma-informed care for PWUD affected by xylazine [51, 52]. Person-first language when referring to people who use xylazine should be widely integrated into clinical training and education programs to mitigate stigma [53, 54].

Civic- and community-based engagement may also serve as promising avenues for combatting xylazine-related stigma in other sectors, including discouraging potentially harmful news media representations of people who have used xylazine. Social and news media also have a significant influence on shaping public attitudes, including framing patients who have used xylazine as ‘zombies’, which may exacerbate stigma and social exclusion [55, 56]. Sensationalist and dehumanizing portrayals of people who use xylazine in the media fuel public stigma and moral panic, diverting attention from complex social and public health factors that increase risk of addiction; lessons should be taken from research on harmful sensationalist news media framing of methamphetamine use [55–57]. Collaborations between public health, medical, and media sectors are essential to set responsible reporting standards, dismantle stigmatizing representations of people who use xylazine, and bridge gaps in access to treatment and harm reduction services.

Lastly, additional research is needed to thoroughly examine the social and structural factors influencing xylazine-involved overdose deaths. Initial findings in the USA suggest that males and non-Hispanic White populations exhibit the highest rates of xylazine-involved overdose deaths, while noteworthy proportions of non-Hispanic Black and Hispanic/Latinx individuals are also affected [27, 28, 31, 34, 35]. Interestingly, these early racial/ethnic trends in xylazine-involved overdoses seem to deviate from the broader landscape of opioid- and IMF-involved overdoses in the USA, for which American Indian and Alaska Native (AI/AN), non-Hispanic Black, and Hispanic/Latinx communities have been disproportionately impacted and are continuing to rise at unequally high rates relative to non-Hispanic White populations [58–62]. Canada, in particular, has a dearth of data regarding racial/ethnic and other sociodemographic differences in xylazine-involved overdose deaths. Further epidemiological and ethnographic research is warranted to investigate these social determinants of health, including regional and temporal variations across North America, to inform harm reduction programs. Xylazine databases should include metrics such as racial/ethnic background, rurality, socioeconomic status, clinical comorbidities, concurrent mental health and substance use disorder diagnoses, and more, to guide public health interventions for medically underserved populations.

### Limitations

There were several limitations. Given the early stage of this emerging public health crisis, a narrative review design was selected for its exploratory approach; however, it did not involve a comprehensive search of the literature. Further, the paucity of research and national-level data on xylazine-involved overdoses in North America limits the representativeness of the spatial and temporal trends highlighted in this study. For instance, these observed trends may partially reflect fundamental differences in individual jurisdictions' epidemiological surveillance and drug checking infrastructure, rather than true shifts in xylazine's involvement in illicit drug supplies and overdoses. Furthermore, the relatively limited number of Canadian studies and data on xylazine-involved overdoses in particular precluded this review from comprehensively assessing the public health impact in Canada. While qualitative and ethnographic studies have shed light on the pathways through which xylazine has infiltrated local unregulated drug markets and supply chains in the USA, similar studies are lacking in Canada. Additional research is warranted to better understand xylazine purchasing, consumption, and overdose patterns, as well as the underlying social and structural determinants of this epidemic. Given the narrative review design and heterogeneity of data sources, graphical visualizations of the epidemiological trends in xylazine-involved overdoses were not included in the present article; however, a systematic review and meta-analysis may be warranted in the future to estimate aggregate frequencies and graphically represent spatiotemporal trends.

## Conclusion

Xylazine is an emerging public health crisis that demands urgent action from multiple spheres of actors—healthcare providers, public health and harm reduction organizations, policymakers, legislators, governments, local and community stakeholders, and others—to address. We have a medical and societal imperative to stem the tide of this crisis and mitigate adverse health outcomes related to xylazine-involved overdoses. Further public health research should be conducted to better understand spatial and temporal trends in such overdoses, as well as the social and structural factors, including unregulated drug market dynamics, that are propelling xylazine’s ongoing spread in North America and globally.

### Supplementary Information

**Additional file 1.** Search strategy.

## Data Availability

Not applicable.
