# Supplementary material for: Public health impact and harm reduction implications of xylazine-involved overdoses: a narrative review
Source: Harm Reduct J. 2023 Sep 12;20:131. doi: 10.1186/s12954-023-00867-x (PMC10498612; doi:10.1186/s12954-023-00867-x)
Supplement: Supplementary file 1 — Additional file 1. Search strategy. [file 12954_2023_867_MOESM1_ESM.docx]

**Table S1.** Narrative review search strategy (conducted on May 28, 2023)

| **Database** | **Search terms** | **Number of results** |
| --- | --- | --- |
| PubMed | (xylazine[tiab] OR Rompum[tiab] OR AnaSed[tiab] OR Sedazine[tiab]) AND (opioid[tiab] OR opiate[tiab] OR overdose[tiab] OR harm reduction[tiab] OR Puerto Rico[tiab] OR Mexico[tiab] OR United States[tiab] OR Canada[tiab]) | 179 |
| Scopus | TITLE-ABS-KEY (xylazine) OR TITLE-ABS-KEY (Rompum) OR TITLE-ABS-KEY (AnaSed) OR TITLE-ABS-KEY (Sedazine) AND TITLE-ABS-KEY (opioid) OR TITLE-ABS-KEY (opiate) OR TITLE-ABS-KEY (overdose) OR TITLE-ABS-KEY (harm reduction) TITLE-ABS-KEY (Puerto Rico) OR TITLE-ABS-KEY (United States) OR TITLE-ABS-KEY (Canada) | 406 |
| Embase | (xylazine and opi*).mp. [mp=title, abstract, heading word, drug trade name, original title, device manufacturer, drug manufacturer, device trade name, keyword heading word, floating subheading word, candidate term word] | 390 |
| Science Direct | xylazine AND opioid AND overdose (Restricted to Review articles (64) Research articles (377) Correspondence (2) Discussion (1) Editorials (1) News (1) Other (33)) | 479 |
| medRxiv | xylazine | 37 |
| bioRxiv | xylazine and opioid or opiate or overdose | 244 |
| Europe PMC | (TITLE:"xylazine" AND ABSTRACT:"opioid" OR ABSTRACT:"opiate" OR ABSTRACT:"xylazine") | 1,264 |
| Total number of articles | 2,999 | |
| Total number of duplicates | 464 | |
| Total after de-depublication | 2,535 | |
